# Fish Hydrolysate Supplementation Prevents Stress-Induced Dysregulation of Hippocampal Proteins Relative to Mitochondrial Metabolism and the Neuronal Network in Mice

**DOI:** 10.3390/foods11111591

**Published:** 2022-05-28

**Authors:** Julie Le Faouder, Bastien Arnaud, Régis Lavigne, Céline Lucas, Emmanuelle Com, Elodie Bouvret, Anne-Laure Dinel, Charles Pineau

**Affiliations:** 1 Abyss Ingredients, 56850 Caudan, France; elodie@abyss-ingredients.com; 2 Protim Core Facility, Biosit UAR 3480 US_S 018, CNRS, Inserm, Rennes University, 35000 Rennes, France; bastien.arnaud@univ-rennes1.fr (B.A.); regis.lavigne@univ-rennes1.fr (R.L.); emmanuelle.com@univ-rennes1.fr (E.C.); charles.pineau@univ-rennes1.fr (C.P.); 3 Irset (Institut de Recherche en Santé, Environnement et Travail)—UMR_S 1085, EHESP, Inserm, Rennes University, 35042 Rennes, France; 4 NutriBrain Research and Technology Transfer, Nutrition et Neurobiologie Intégrée, UMR 1286, 33076 Bordeaux, France; celine.lucas@nutribrain.fr (C.L.); anne-laure.dinel@inrae.fr (A.-L.D.); 5 NutriNeuro, INRAE, Bordeaux INP, Bordeaux University, 33076 Bordeaux, France

**Keywords:** fish hydrolysate, proteomics, acute mild stress, mouse, hippocampus, mitochondria, neuronal network

## Abstract

Over the past several decades, stress has dramatically increased in occidental societies. The use of natural resources, such as fish hydrolysates, may be an attractive strategy to improve stress management. Our previous study demonstrated the anxiolytic effects of fish hydrolysate supplementation in mice exposed to acute mild stress by limiting stress-induced corticosterone release and modulating the expression of a number of stress-responsive genes. Here, we explore hippocampal protein modulation induced by fish hydrolysate supplementation in mice submitted to acute mild stress, with the aim of better elucidating the underlying mechanisms. Hippocampi from the same cohort of Balb/c mice supplemented with fish hydrolysate (300 mg·kg^−1^ body weight) or vehicle daily for seven days before being submitted or not to an acute mild stress protocol (four groups, *n* = 8/group) were subjected to label-free quantitative proteomics analysis combined with gene ontology data mining. Our results show that fish hydrolysate supplementation prevented the observed stress-induced dysregulation of proteins relative to mitochondrial pathways and the neuronal network. These findings suggest that fish hydrolysate represents an innovative strategy to prevent the adverse effects of stress and participate in stress management.

## 1. Introduction

Stress has dramatically increased in occidental societies over the past several decades [1]. Stress triggers the activation of the hypothalamo–pituitary–adrenal (HPA) axis, which regulates circulating levels of glucocorticoid hormones (i.e., cortisol in humans and corticosterone in rodents) and represents the major neuroendocrine axis that regulates homeostasis in mammals [2]. Glucocorticoids are released by the adrenal cortex in response to stressful environmental changes. They act both on peripheral organs and the brain, affecting metabolism, immunity, neurotransmission, excitotoxicity, neuroplasticity, behavior, emotions, and cognition [3]. When persistent and left untreated, stress can result in serious health problems, such as depression, burnout, anxiety, and sleep disorders [1].

The use of natural resources, such as fish hydrolysates, may be an attractive strategy to improve stress management. They are generally produced from fish by-products, thus minimizing the economic and environmental cost [4]. Protein hydrolysis improves the functional properties of the product by allowing the release of low molecular-weight peptides with several bioactivities, such as antioxidant, antimicrobial, antihypertensive, anti-inflammatory, antihyperglycemic, skin anti-aging, and anxiolytic effects, among others [5,6,7].

Belhaj et al. demonstrated the anxiolytic-like and neuroprotective effects of a phospholipopeptidic complex obtained by the enzymatic hydrolysis of salmon heads in mice [8]. Chataigner et al. showed that a fish hydrolysate containing n-3 long chain polyunsaturated fatty acids (n-3 LC-PUFAs) and low molecular-weight peptides displayed anxiolytic activities, with the reduction of anxiety-like behavior in aged mice, the restoration of plasma corticosterone levels similar to those in adult animals following acute stress, and the modulation of hypothalamic stress-responsive gene expression [9]. In rats, Bernet et al. demonstrated the diazepam-like effect of fish protein hydrolysate supplementation (1.2 mg·kg^−1^) on stress responsiveness [10], whereas Messaoudi et al. demonstrated the potential anxiolytic- and antidepressant-like properties of a protein autolysate from the blue ling fish, in the absence of any changes in cerebral activation or dependence [11]. More recently, Freret et al. showed that both the natural products, αS1–casein hydrolysate and fish hydrolysate (oral dose of 15 mg·kg^−1^), were as efficient as diazepam in reducing anxiety levels in rats, along with fast-acting anxiolytic activity of the fish hydrolysate [12]. Of note, the results of a study by Messaoudi et al. suggested the antistress activity of αS1-casein hydrolysate in human subjects based on changes in blood pressure and cortisol levels, confirming the interest in natural hydrolysates for stress management [13].

In a previous study, we demonstrated that short-term supplementation (7 days) with a low dose of fish hydrolysate (300 mg·kg^−1^) had anxiolytic-like activity in mice exposed to acute mild stress, notably by limiting stress-induced corticosterone release [14]. We evaluated the effect of fish hydrolysate on the expression of 93 stress-responsive genes in several brain structures involved in mood (pre-frontal cortex, amygdala, and hippocampus). Most of the regulated genes are involved in the regulation of the HPA axis or in mitochondrial activity, which are essential for stress management. Fish hydrolysate may also modulate circadian rhythms and the aging process. Moreover, the hippocampus appears to be a structure that is highly modulated by fish hydrolysate supplementation in the context of acute mild stress. Interestingly, the hippocampus is classically described to be a brain area highly involved in mood regulation [15]. It is well known that proteins are the main executors of physiological functions in organisms. Thus, their quantitative analysis can help improve our understanding of pathophysiological mechanisms and the actions of their treatment. Here, we applied a label-free quantitative proteomics approach to explore the modulation of hippocampal proteins induced by fish hydrolysate supplementation in mice exposed to an acute mild stress. By preventing stress-induced dysregulation of proteins involved in mitochondrial pathways and the neuronal network, fish hydrolysate supplementation appears to be an innovative strategy to regulate and manage stress.

## 2. Materials and Methods

### 2.1. Fish Hydrolysate

Peptidyss^®^ is a water-soluble powder obtained from the standardized enzymatic hydrolysis of sardine by-products without preservatives or processing aids. Its natural composition is specific, containing more than 70% peptides (Table 1 and Table 2), of which 98% have a molecular weight under 3000 Da and 50% under 500 Da.

### 2.2. Experimental Design 

**Figure 1:**
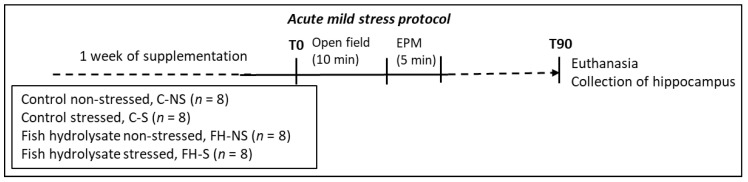
Experimental protocol for acute mild stress in adult Balb/c mice.

Experimental design described in [14] is summarized in the supplementary materials and methods file. Acute mild stress was induced by submitting the mice to an open field (OF) test for 10 min immediately followed by an elevated plus maze (EPM) test for 5 min (Figure 1).

### 2.3. Label-Free Quantitative Proteomics 

#### 2.3.1. Protein Extraction

Proteins were recovered after RNA isolation with TRIzol^®^ from mouse hippocampi using the protocol of Simoes et al. [16]. The protein concentration was determined using the bicinchoninic acid protein assay (Interchim, Montluçon, France) according to the manufacturer’s instructions.

#### 2.3.2. Nanoliquid Chromatography Coupled with Tandem Mass Spectrometry (NanoLC-MS/MS)

Samples were subjected to reduction, alkylation, digestion, and purification. Ten micrograms of protein were reduced by adding 2.5 µL 65 mM dithiothreitol in 50 mM ammonium bicarbonate, with incubation at 37 °C for 15 min. Alkylation was performed by adding 2.5 µL 135 mM iodoacetamide and incubating for 15 min at room temperature in the dark. Proteins were first digested by adding 2 µL Lys-C at 0.1 µg·µL^−1^ (Promega, Madison, WI, USA) in 23 µL 50 mM ammonium bicarbonate with 0.01% ProteaseMAX surfactant (Promega) with incubation at 37 °C for 4 h. Then, proteins were digested by adding 4 µL trypsin (sequencing grade modified, Promega) at 0.1 µg·µL^−1^ in 46 µL 50 mM ammonium bicarbonate with 0.01% ProteaseMAX and incubated at 37 °C overnight. Samples were then purified from salts, contaminants, and detergents using Phoenix cartridges (PreOmics GmbH, Martinsried, Germany).

Approximately 200 ng of the resulting peptide mixtures were analyzed by nanoLC-MS/MS using the protocol of Méar et al. [17] also detailed in the supplementary materials and methods file.

#### 2.3.3. Quantification and Statistical Analyses of Proteomics Data

All MS/MS data were processed using MaxQuant (version 1.6.17; Max-Planck-Institute of Biochemistry, Martinsried, Germany). The precursor ions mass tolerances were set to 20 and 10 ppm for the first and main searches, respectively. A fragment tolerance of 20 ppm was applied. Oxidation (M) and acetylation (protein N-term) were allowed for variable modifications and carbamidomethylation (C) for the fixed modification. The maximum number of missed cleavages by trypsin was limited to two. MS/MS data were analyzed using the extracted database (2021) from UniProt with the *Mus musculus* taxonomy (55,315 entries). Peptide-spectrum matches (PSMs) and protein identifications were validated using a false discovery rate (FDR) threshold of 1%. Differences in peptide and protein abundance were measured using the fast label free quantitation (LFQ) algorithm with a minimum ratio count of 2, classic data normalization, and the match between run option. Unmodified unique and razor peptides were used for each protein quantification.

Statistical analyses of proteomic experiments were performed using Perseus software (version 1.6.15; Max-Planck-Institute of Biochemistry, Martinsried, Germany). Missing values were imputed from a normal distribution with a width of 0.3 and a down-shift parameter of 1.8. A two-way ANOVA test, with supplementation and stress as factors, was applied. The three generated lists of differential proteins for stress, supplementation and interaction Stress × Supplementation (*p* < 0.05) were independently classified using unsupervised Bayesian clustering [18]. Proteins were classified according to their expression among the four groups, and each generated cluster was used separately for the analysis of gene ontology (GO) term enrichment. The Cytoscape plug-in Bingo [19] was used with the following parameters: right-sided hypergeometric test with a Benjamini–Hochberg correction, a p-value threshold of 0.05, and the use of all quantified proteins as the reference set. The visualization of enriched gene ontology terms was conducted following the protocol described by [20]. For protein annotation, the entire annotation from *Mus musculus* was used as the reference set. Principal component analysis (PCA) and sparse partial least squares discriminant analysis (sPLS-DA) as well as box plots were performed using the MetaboAnalyst 5.0 web server [21].

## 3. Results

### 3.1. Hippocampal Protein Expression Is Modulated by Stress, Supplementation, and the Interaction Stress × Supplementation

**Figure 2:**
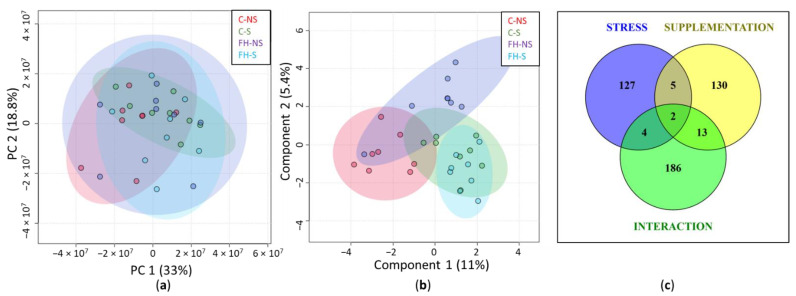
Evaluation of quantified hippocampal proteins (*n* = 4149). (**a**) Graphic representation defined by the first two principal components (PC1 and 2) of the principal component analysis (PCA). (**b**) Graphic representation defined by the first two components (1 and 2) of the sparse partial least squares discriminant analysis (sPLS-DA). (**c**) Venn diagram representing the differential proteins (*p* < 0.05) for stress, supplementation, and interaction of Stress × Supplementation after two-way ANOVA analysis.

In the overall series (*n* = 32), 5166 proteins were quantified from mouse hippocampus. Among them, 4149 proteins with at least two identified peptides were further considered (Supplementary Table S1). PCA of the quantified proteins showed no sampling problems: samples were relatively scattered, with limited variation between groups, as expected for brain proteins, following the acute mild stress protocol (Figure 2a). The sPLS-DA of quantified proteins showed the contribution of stress to component 1 and supplementation to component 2 (Figure 2b). This result shows that the expression of hippocampal proteins was modulated by both stress and supplementation factors.

Two-way ANOVA showed 138, 150, and 205 differential proteins (*p* < 0.05) for stress, supplementation, and the interaction Stress × Supplementation, respectively, with only a few proteins common to the three groups (Figure 2c). This result not only confirmed the separate effect of stress and supplementation on protein expression, but also show the effect of the interaction between stress and supplementation.

### 3.2. Stress Modulates the Expression of Proteins Involved in Dopamine Metabolism, Neuron Projection, and Metabolic Processes

**Figure 3:**
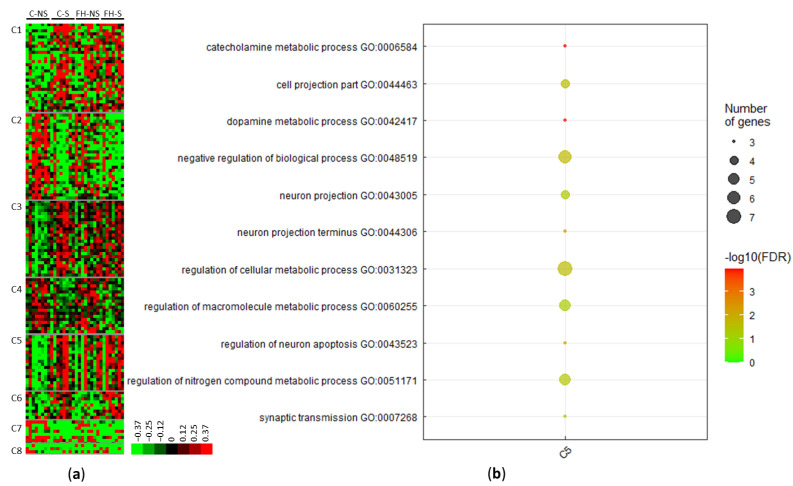
Proteins differentially expressed with acute mild stress (*n* = 138). (**a**) Unsupervised Bayesian clustering for the four groups (*n* = 8/group): control non-stressed (C-NS), control stressed (C-S), fish hydrolysate non-stressed (FH-NS), fish hydrolysate stressed (FH-S). (**b**) Gene ontology (GO) term enrichment for proteins upregulated with stress from cluster C5.

Clustering of the 138 stress-related differential proteins revealed eight clusters (C1–C8) (Figure 3a, Supplementary Table S2). Four clusters included proteins for which the levels increased following acute mild stress (i.e., C1, C3, C5, and C6). Proteins in cluster C1 matched with the terms: catalytic activity (10/25; e.g., mitogen-activated protein kinase 8, Mapk8), purine ribonucleotide binding (7/25; e.g., Ras-related protein Rab-31, Rab31), mitochondrion (6/25; e.g., 28S ribosomal protein S7, Mrps7; NADH dehydrogenase [ubiquinone] 1 alpha subcomplex assembly factor, Ndufaf4; 28S ribosomal protein S29, Dap3, Mrps29), and ligase activity (4/25; e.g., E3 ubiquitin-protein ligase LRSAM1, Lrsam1).

Proteins in cluster C3 matched with the terms catalytic activity (14/23; e.g., S-phase kinase-associated protein 1, Skp1), metabolic processes (12/23; e.g., serine/threonine-protein kinase PAK 2, Pak2), transport (7/23; e.g., V-type proton ATPase subunit G1, Atp6v1g1), mitochondrion (6/23; e.g., superoxide dismutase [Mn], Sod2), transferase activity (5/23; e.g., nucleoside diphosphate kinase B, Nme2), and neuron projection (3/23; e.g., protein RUFY3, Rufy3).

Proteins upregulated in cluster C5 corresponded to enrichment in pathways, such as dopamine metabolic processes (3/14, 21.4%, *p* = 1.0662 × 10^−4^), neuron projection (4/14, 28.5%, *p* = 4.4413 × 10^−2^), and the regulation of macromolecule metabolic processes (5/14, 35.7%, *p* = 4.8267 × 10^−2^) (Figure 3b, Supplementary Table S2). These pathways and the associated proteins are presented in Table 3.

Three clusters brought together proteins with decreased expression following acute mild stress (C2, C4, and C7) (Supplementary Table S2). Proteins upregulated in cluster C2 matched with the terms: metabolic process (14/26, e.g., adenylate cyclase type 5, Adcy5), catalytic activity (12/26, e.g., NT-3 growth factor receptor, Ntrk3), and purine nucleotide binding (8/26, e.g., SNF-related serine/threonine-protein kinase, Snrk). Proteins upregulated in cluster C4 matched with the terms: metabolic process (10/14, e.g., cAMP-dependent protein kinase type I-beta regulatory subunit, Prkar1b), developmental process (6/14, e.g., SWI/SNF complex subunit SMARCC2, Smarcc2), transport (5/14, e.g., NADH dehydrogenase [ubiquinone] 1 beta subcomplex subunit 9, Ndufb9), and dendrite (2/14, e.g., catenin delta-1, Ctnnd1).

### 3.3. Fish Hydrolysate Supplementation Modulates the Expression of Proteins Involved in Metabolic Processes and the Neuronal Network

**Figure 4:**
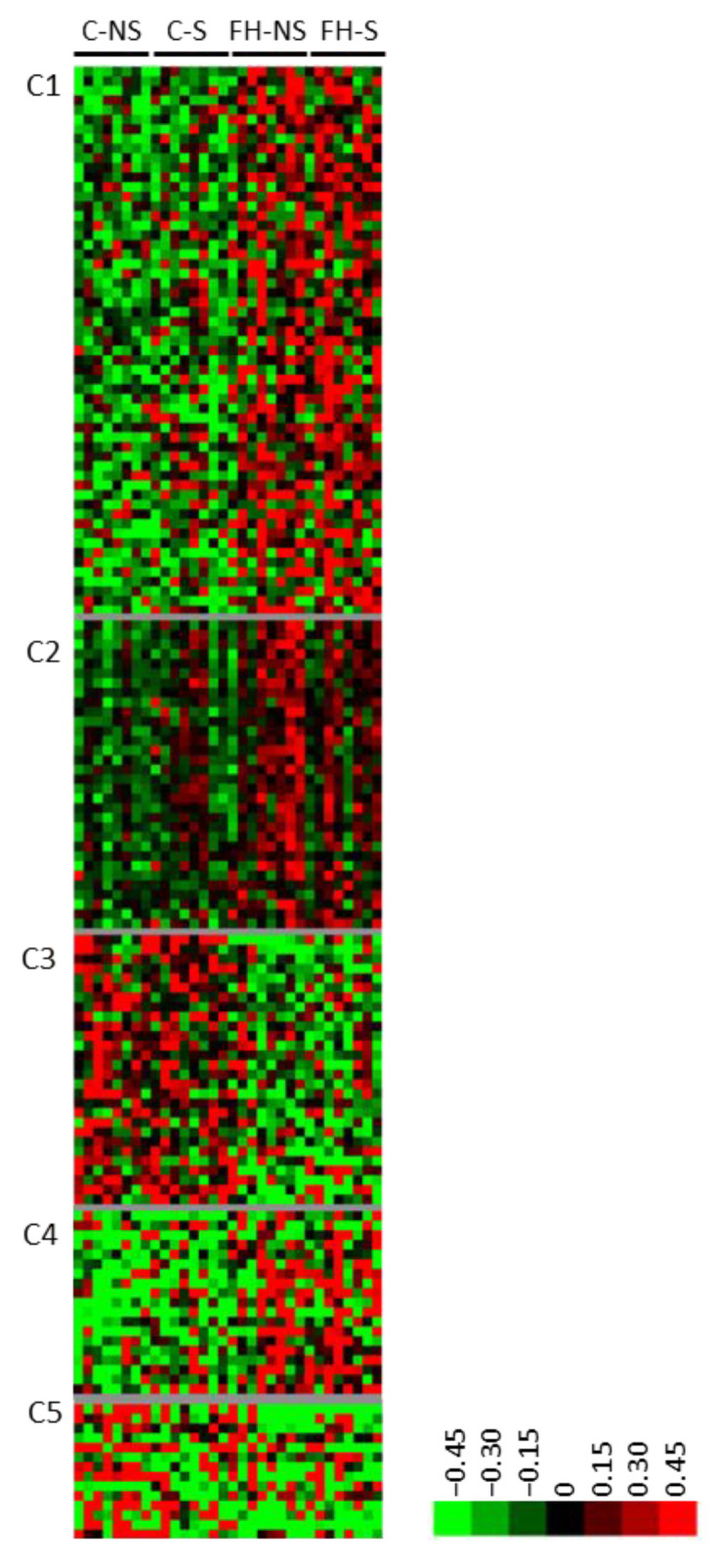
Proteins differentially expressed with supplementation (*n* = 150). Unsupervised Bayesian clustering for the four groups (*n* = 8/group).

Clustering of the 150 supplementation-related differential proteins showed five clusters (C1-C5) (Figure 4, Supplementary Table S3). Three clusters corresponded to proteins with increased expression following supplementation (C1, C2 and C4). Several proteins from cluster C1 are involved in hydrolase (9/42, e.g., pyrroline-5-carboxylate reductase 3, Pycr3; ATP-dependent DNA/RNA helicase DHX36, Dhx36) or pyrophosphatase (6/42, e.g., RuvB-like 1, Ruvbl1) activities, purine ribonucleotide binding (8/42, e.g., Rho-related GTP-binding protein RhoQ), and apoptosis (5/42, e.g., sequestosome-1, Sqstm1). Several proteins from cluster C2 are involved in the neuronal network (4/26, tubulin-specific chaperone E, Tbce; 14-3-3 protein eta, Ywhah; platelet-activating factor acetyl hydrolase IB subunit beta, Pafah1b1; and guanine nucleotide-binding protein G(q) subunit alpha, Gnaq). Several proteins from cluster C4 are involved in cell-junction organization (2/18, coxsackievirus and adenovirus receptor homolog, Cxadr and Merlin, Nf2). Two clusters regrouped proteins for which the expression decreased following supplementation (C3 and C5). Several proteins in cluster C3 are involved in metabolic processes (12/21; e.g., acyl-coenzyme A thioesterase 11, Acot11), catalytic activity (10/21; e.g., NADH-cytochrome b5 reductase 3, Cyb5r3), purine ribonucleotide binding (6/21; e.g., heat shock protein 75 kDa, mitochondrial, Trap1), and mitochondria (5/21; e.g., mitochondrial 2-oxoglutarate/malate carrier protein, Slc25a11).

### 3.4. Fish Hydrolysate Supplementation Prevents Stress-Induced Dysregulation of Proteins Relative to Mitochondrial Pathways and the Neuronal Network

**Figure 5:**
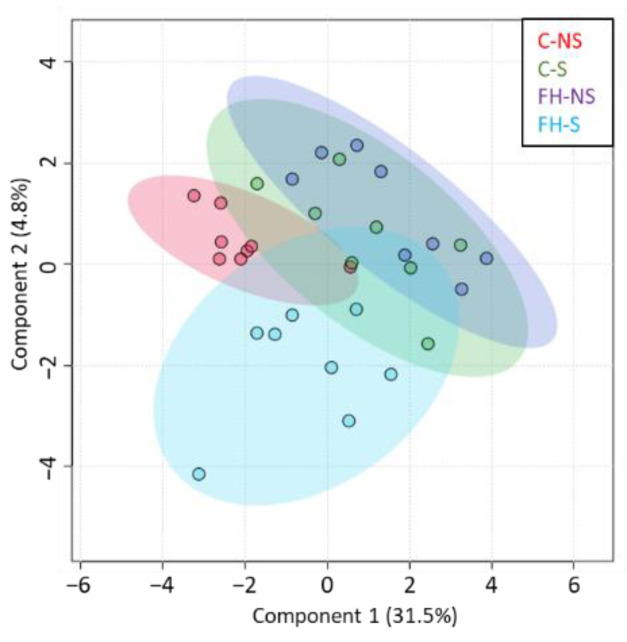
Proteins differentially expressed with the interaction Stress × Supplementation (*n* = 205). Graphic representation defined by the first two components (1 and 2) of the sparse partial least squares discriminant analysis (sPLS-DA).

The sPLS-DA of differential proteins from the interaction supplementation × stress (*n* = 205) showed that the expression of proteins in stressed animals supplemented with fish hydrolysate was different from that of the other groups (Figure 5).

**Figure 6:**
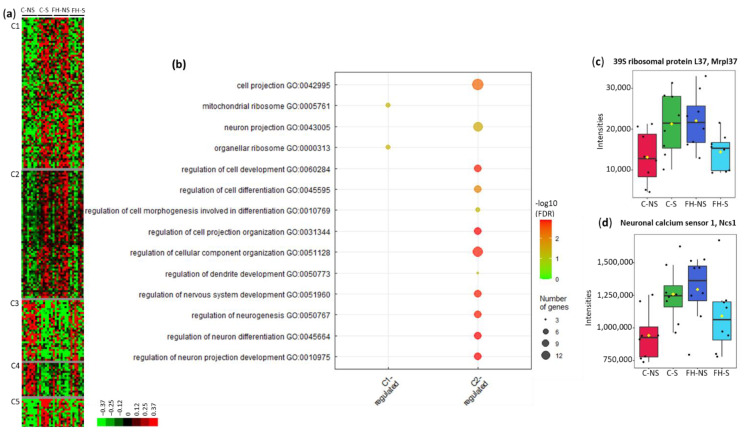
Proteins differentially expressed with the interaction Stress × Supplementation (*n* = 205). (**a**) Unsupervised Bayesian clustering for the four groups (*n* = 8/group). (**b**) Gene ontology (GO) term enrichment from clusters C1 and C2. (**c**) Box plots of 39S ribosomal protein L37 from cluster C1. (**d**) Box plots of neuronal calcium sensor 1 from cluster C2.

Clustering of these proteins led to five clusters (C1–C5) (Figure 6a, Supplementary Table S4). In clusters C1, C2 and C5, protein levels were upregulated following stress in control animals and downregulated following stress in supplemented animals. Gene ontology analysis on proteins from cluster C1 showed an enrichment in biological pathways related to organellar ribosomes and mitochondrial ribosomes (4/54, 7.4%, *p* = 4.3287 × 10^−2^) (Figure 6b). The intensities of 39S ribosomal protein L37 within the four groups are displayed in Figure 6c. In cluster C1, 24/54 (44.4%) proteins are involved in metabolic processes: 11 mitochondrial proteins, 7 proteins from oxidation reduction, and 6 from lipid metabolism processes (Table 4, Supplementary Table S4).

Gene ontology analysis of proteins in cluster C2 showed enrichment in biological pathways related to the neuronal network (e.g., regulation of neuron projection development (7/50, 14.0%, *p* = 1.2572 × 10^−3^) and regulation of cellular component organization (11/50, 22.0%, *p* = 1.3827 × 10^−3^) (Figure 6b, Supplementary Table S4). These pathways and associated proteins are presented in Table 5. The intensities of neuronal calcium sensor 1 within the four groups are displayed in Figure 6d.

Cluster C2 also included several proteins associated with transport (16/50; e.g., sodium/potassium-transporting ATPase subunit alpha-1, Atp1a1), the regulation of metabolic processes (11/50; e.g., glycogen synthase kinase-3 beta, Gsk3b), purine ribonucleotide binding (8/50; e.g., Ras-related protein Rab-21, Rab21), and the regulation of catalytic activity (6/50; e.g., PEX5-related protein, Pex5l) (Supplementary Table S4).

The expression of proteins in clusters C3 and C4 was upregulated following stress in supplemented animals and downregulated following stress in control animals. Cluster C3 included proteins associated with purine ribonucleotide binding (6/26, e.g., potassium/sodium hyperpolarization-activated cyclic nucleotide-gated channel 2, Hcn2), small molecule metabolic processes (5/26, e.g., Ras-related protein Rab-5C, Rab5c), the regulation of catalytic activity (4/26, e.g., adenylate cyclase type 1, Adcy1), and phosphorus-oxygen lyase activity (2/26, e.g., guanylate cyclase soluble subunit alpha-1, Gucy1a1). Cluster C4 included proteins associated with cellular metabolic processes (7/11, e.g., fatty acid CoA ligase Acsl3, Acsl3), catalytic activities (6/11, e.g., serine/threonine-protein phosphatase PGAM5, mitochondrial, Pgam5), synapses (3/11, e.g., calcium/calmodulin-dependent protein kinase type II subunit delta, Camk2d), and the response to stress (3/11, e.g., phosphatidylinositol 3-kinase catalytic subunit type 3, Pik3c3).

## 4. Discussion

In this study, we performed label-free quantitative proteomic analysis to explore hippocampal protein modulation induced by fish hydrolysate supplementation in mice exposed to an acute mild stress.

In a previous study, we confirmed the induction of stress by an increase in corticosterone secretion and modulation of the expression of stress responsive genes, mostly those involved in the HPA axis pathway [14]. Here, we show that such stress, although moderate, also led to the modulation of several hippocampal proteins. Acute mild stress induced the translation of proteins involved in mood. Indeed, stress induced the upregulation of α- and β-synucleins, proteins, involved in dopamine (DA) metabolism. DA is a neurotransmitter that plays an important role in cognitive and emotional regulation. It is a precursor of noradrenaline, a stress hormone synthesized during stressful events. Although the role of α- and β-synucleins is still unclear, they are likely involved in neuronal growth and plasticity, and the transport and release of DA [22]. These results are in accordance with those of Chiavegatto et al., who reported increased α-synuclein mRNA and protein expression, together with decreased dopaminergic activity, in the hippocampus of anxious rats relative to less-anxious rats [23]. β-synuclein was shown by Carboni et al. to be protein upregulated after repeated exposure to psychosocial stress in the rat hippocampus [24]

As previously demonstrated, fish hydrolysate supplementation modulated the expression of stress-responsive genes involved in regulation of the HPA axis, as well as genes involved in mitochondrial metabolism, the circadian rhythms, and the aging process [14]. Here, we show that fish hydrolysate supplementation also modulated the translation of several proteins principally involved in metabolic processes and neuronal networks. Interestingly, fish hydrolysate supplementation induced the translation of 14-3-3 protein eta (Ywhah), which is particularly involved in the regulation of several key neuronal processes, such as neurotransmission, dendritic complexity, and spine density [25]. Fish hydrolysate supplementation could thus promote the neuronal network and facilitate the response of the central nervous system to aversive events, such as stress.

Above all, we show that fish hydrolysate supplementation prevented stress-induced dysregulation of hippocampal proteins relative to mitochondrial metabolism and the neuronal network. First, fish hydrolysate supplementation downregulated several mitochondrial ribosomal proteins (MRPs) (i.e., 39S ribosomal protein L40, Mrpl40; 39S ribosomal protein L30, Mrpl30; 39S ribosomal protein L37, Mrpl37; and 28S ribosomal protein S29, Dap3). MRPs are encoded by nuclear genes and synthesized by cytoplasmic 80S ribosomes after specific targeting, sorting, and transport to the mitochondria. Each MRP is essential for the composition of the mitochondrial ribosome, which plays an irreplaceable role in the assembly and translation of mitochondrial DNA. Although their role has not yet been clearly elucidated, MRPs appear to be key proteins in the process of apoptosis. Abnormal expression of MRPs leads to mitochondrial metabolism disorders, cell dysfunction, etc. [26]. Recent studies also showed that MRPs are positively associated with cognitive function in elderly women [27].

Fish hydrolysate supplementation also downregulated other mitochondrial proteins, such as NADH dehydrogenase [ubiquinone], flavoprotein 3 (Ndufv3), and ATP synthase F(0) complex subunit B1 (Atp5pb). In our previous study, we highlighted an interaction between supplementation and stress for the mitochondrial genes ATP8, ATP6, and COX1 [14]. Mitochondria are endocrine organelles that provide both the energy and signals that enable and direct the adaptation to stress [28]. Alterations of mitochondrial metabolism and oxidative stress are observed in high anxiety. Conversely, it was previously demonstrated that changes in mitochondrial function can also lead to heightened anxiety [29]. Tang et al. demonstrated that chronic mild stress modulates the expression of mitochondrial and metabolic proteins in the rat hippocampus by proteomic analysis [30]. Misiewicz et al. identified mitochondrial pathways associated with anxiety-related behavior in mice by multi-omics analyses [31]. In addition, several studies confirmed the importance of mitochondrial signaling during stress [28]. Fish hydrolysate, by inhibiting mitochondrial alterations, appears to be an attractive solution to manage stress.

Fish hydrolysate supplementation downregulated several proteins involved in the neuronal network, e.g., nesprin-1 (Syne 1), neuronal calcium sensor 1 (Ncs1), glycogen synthase kinase-3 beta (Gsk3b), and kalirin (Klrn). Interestingly, these proteins all contribute to the integrative organization of the neuronal network. Nesprin-1 (Syne 1) has been shown to play critical roles in neurogenesis and neuronal migration in mice [32]. Neuronal calcium sensor 1 (Ncs1) was reported to modulate neuronal morphology and development [33]. Glycogen synthase kinase-3 beta (Gsk3b) inhibition activates neurogenesis [34]. Kalirin is known to play a key role in synaptic plasticity and the formation of dendritic trees and spines [35]. Chronic stress has been shown to exert detrimental effects on hippocampal neurogenesis and neuroplasticity in patients and stress-induced animal models [36,37], consequently leading to cognitive and emotional symptoms of depression and anxiety [38]. By modulating adult hippocampal neurogenesis, fish hydrolysate could prevent the alterations induced by stress and may be an attractive solution to manage stress.

Most of these proteins are also key factors in the regulation of the DA pathway. Ncs1 has been found to regulate the phosphorylation, trafficking, and signaling profile of the receptor of type 2 dopaminergic neurons (D2R) [39]. DA regulates mitochondrial motility in cultured hippocampal neurons through the Akt-GSK3beta signaling cascade [40] and kalirin may be directly regulated through D2R postsynaptic neurons [41]. Moreover, it was shown that the elimination of kalirin expression in proopiomelanocortin cells reduces anxiety-like behavior and contextual fear learning [42].

Overall, those results suggest that fish hydrolysate supplementation could prevent the deleterious effects of acute mild stress by modulating the DA pathway. However, the interaction between fish hydrolysate and the DA pathway requires further investigation.

Finally, our previous study showed that the expression of Per2, one of the most important circadian rhythm genes, was increased by stress and supplementation in the hippocampus [14]. Here, we show that fish hydrolysate supplementation prevented the stress-induced increase of proteins associated with regulation of the circadian rhythm pathway, i.e., the serine/threonine-protein phosphatase PP1-gamma catalytic subunit (Ppp1cc) and glycogen synthase kinase-3 beta (Gsk3b). Protein phosphatase 1 (PP1) has been shown to be a post-translational regulator of the mammalian circadian clock [43], while glycogen synthase kinase 3 (GSK3) activity was shown to regulate rhythms in hippocampal clock gene expression and synaptic plasticity [44]. Glycogen synthase kinase 3 beta has been shown to alter anxiety-, depression-, and addiction-related behaviors in rats, as well as neuronal activity in the shell of the nucleus accumbens [45]. Overall, these results confirm the anxiolytic-like activity of fish hydrolysate supplementation and suggest a potential role in the regulation of circadian rhythms that should be further investigated.

Our study had several limitations. An acute mild stress protocol and short supplementation period (i.e., one week) undoubtedly induced limited modulation of protein expression in the hippocampus. The high sensitivity of the mass spectrometer used for our proteomics analyses allowed us to analyze several low-copy number differential proteins, resulting, however, in a moderate enrichment in GO terms with sometimes few implicated proteins. Thus, obtained results would require further validation on an independent cohort. Likewise, chronic stress models with longer supplementation periods should confirm the mechanisms underlying the better reactivity to stress induced by fish hydrolysate supplementation assessed in this study and eventually provide additional insights into other molecular mechanisms. A complementary metabolomics approach could be relevant for further validating the role of fish hydrolysate in regulating stress by demonstrating its involvement in pathways related to metabolic processes. Finally, characterization of the peptides from fish hydrolysate responsible for its anxiolytic-like activity is ongoing in our laboratory.

## 5. Conclusions

In this study, we applied label-free quantitative proteomics analysis to obtain a comprehensive picture of the mouse hippocampal response to acute mild stress after fish hydrolysate supplementation. Our results demonstrate that fish hydrolysate rescues the adverse effects of mild stress by regulating mitochondrial pathways and the neuronal network, which are essential for stress management. Fish hydrolysate appears to be an innovative strategy to regulate and manage stress. Moreover, fish hydrolysate may modulate circadian rhythms. Complementary studies are required to reinforce these preliminary data and investigate the role of fish hydrolysate in diseases associated with aging, such as neurodegenerative diseases, which are tightly associated with alterations in the circadian rhythms.

## Figures and Tables

**Table 1:** Total composition of fish hydrolysate supplementation.

Composition	Fish Hydrolysate
Total proteins	≥70%
Minerals	≤20%
Lipids	≤5%

**Table 2:** Amino-acid composition of fish hydrolysate supplementation.

Composition of Amino Acids	Fish Hydrolysate
Essential Amino Acids	43%
Non-essential Amino Acids	56%
Branched-Chain Amino Acids	17%
Sulfur Amino Acids	3%

**Table 3:** Pathways and associated proteins upregulated by acute mild stress.

Pathway Name	Protein IDs	Protein Name	Gene Name	Expression C-S/FH-S
Dopamine metabolic	P00493	Hypoxanthine-guanine phosphoribosyltransferase	Hprt1	up
Process *	Q91ZZ3	Beta-synuclein	Sncb	up
	O55042	Alpha-synuclein	Snca	up
Neuron projection *	Q08460	Calcium-activated potassium channel subunit alpha-1	Kcnma	up
	A2A8L5	Receptor-type tyrosine-protein phosphatase F	Ptprf	up
	Q91ZZ3	Beta-synuclein	Sncb	up
	O55042	Alpha-synuclein	Snca	up
Regulation	Q9EQQ9	Protein O-GlcNAcase	Oga	up
of macromolecule metabolic	Q8CI32	BAG family molecular chaperone regulator 5	Bag5	up
Process *	P29595	NEDD8	Nedd8	up
	O55042	Alpha-synuclein	Snca	up
	Q9Z1X4	Interleukin enhancer-binding factor 3	Ilf3	up

* significant pathway (*p* < 0.05).

**Table 4:** Proteins from mitochondrial pathways modulated by the interaction Stress × Supplementation.

Pathway Name	Protein IDs	Protein Name	Gene Name	Expression FH-S vs. C-S
Mitochondrial	Q9Z2Q5	39S ribosomal protein L40	Mrpl40	down
Ribosome *	Q9D7N6	39S ribosomal protein L30	Mrpl30	down
	Q921S7	39S ribosomal protein L37	Mrpl37	down
	Q9ER88	28S ribosomal protein S29	Dap3	down
Mitochondrion	P09528	Ferritin heavy chain	Fth1	down
	Q8BK30	NADH dehydrogenase [ubiquinone] flavoprotein 3	Ndufv3	down
	Q9Z2Q5	39S ribosomal protein L40	Mrpl40	down
	Q9CZL5	Pterin-4-alpha-carbinolamine dehydratase 2	Pcbd2	down
	Q9CQQ7	ATP synthase F(0) complex subunit B1	Atp5pb	down
	Q9D7N6	39S ribosomal protein L30	Mrpl30	down
	Q921S7	39S ribosomal protein L37	Mrpl37	down
	Q9ER88	28S ribosomal protein S29	Dap3	down
	Q9CR21	Acyl carrier protein	Ndufab1	down
	Q9CQX8	28S ribosomal protein S36	Mrps36	down
	P62897	Cytochrome c, somatic	Cycs	down
Oxidation	P09528	Ferritin heavy chain	Fth1	down
Reduction	Q8BK30	NADH dehydrogenase [ubiquinone] flavoprotein 3	Ndufv3	down
	Q9CQF9	Prenylcysteine oxidase	Pcyox1	down
	Q8BGW1	Alpha-ketoglutarate-dependent dioxygenase FTO	Fto	down
	Q9CR21	Acyl carrier protein	Ndufab1	down
	Q8K0C4	Lanosterol 14-alpha demethylase	Cyp51a1	down
	P62897	Cytochrome c, somatic	Cycs	down
Lipid metabolism	Q6P549	Phosphatidylinositol 3,4,5-trisphosphate 5-phosphatase 2	Inppl1	down
	Q80Y98	Phospholipase DDHD2	Ddhd2	down
	Q9CR21	Acyl carrier protein	Ndufab1	down
	Q8K0C4	Lanosterol 14-alpha demethylase	Cyp51a1	down
	Q00915	Retinol-binding protein 1	Rbp1	down
	Q9D2R0	Acetoacetyl-CoA synthetase	Aacs	down

* significant pathway (*p* < 0.05).

**Table 5:** Proteins from the neuronal network modulated by the interaction Stress × Supplementation.

Pathway Name	Protein IDs	Protein Name	Gene Name	Expression FH-S vs. C-S
Regulation of	A2CG49	Kalirin	Kalrn	down
Neuron	Q9WV60	Glycogen synthase kinase-3 beta	Gsk3b	down
Projection	P08553	Neurofilament medium polypeptide	Nefm	down
Development *	Q99P72	Reticulon-4	Rtn4	down
	Q8BNY6	Neuronal calcium sensor 1	Ncs1	down
	Q6ZWR6	Nesprin-1	Syne1	down
	Q5SNZ0	Girdin	Ccdc88a	down
Regulation of	Q61301	Catenin alpha-2	Ctnna2	down
Cellular	A2CG49	Kalirin	Kalrn	down
Component	Q9WV60	Glycogen synthase kinase-3 beta	Gsk3b	down
Organization *	P08553	Neurofilament medium polypeptide	Nefm	down
	P27546	Microtubule-associated protein 4	Map4	down
	P47757	F-actin-capping protein subunit beta	Capzb	down
	Q99P72	Reticulon-4	Rtn4	down
	P70336	Rho-associated protein kinase 2	Rock2	down
	Q8BNY6	Neuronal calcium sensor 1	Ncs1	down
	Q6ZWR6	Nesprin-1	Syne1	down
	Q5SNZ0	Girdin	Ccdc88a	down

* significant pathway (*p* < 0.05).

## Data Availability

The mass spectrometry proteomics data have been deposited at the ProteomeXchange Consortium via the PRIDE [46] partner repository with the dataset identifier PXD033053.

## Supplementary Materials

The following supporting information can be downloaded at: https://www.mdpi.com/article/10.3390/foods11111591/s1 Table S1: List of quantified proteins after MaxQuant processing and Perseus data analyses, four groups (*n* = 8/group); Table S2: Proteins differentially expressed with acute mild stress. Repartition of these proteins among clusters (C1–C8). Enriched GO terms and associated proteins from cluster C5; Table S3: Proteins differentially expressed with supplementation. Repartition of these proteins among clusters (C1–C5); Table S4: Proteins differentially expressed with the interaction Stress × Supplementation. Repartition of these proteins among clusters (C1–C5). Enriched GO terms and associated proteins from clusters C1 and C2. File S1: Meterial and Methods.
