# Supplementary material for: Fish Hydrolysate Supplementation Prevents Stress-Induced Dysregulation of Hippocampal Proteins Relative to Mitochondrial Metabolism and the Neuronal Network in Mice
Source: Foods. 2022 May 28;11(11):1591. doi: 10.3390/foods11111591 (PMC9180483; doi:10.3390/foods11111591)
Supplement: Supplementary file 1 [file foods-11-01591-s001.zip › File S1-materials-and-methods.pdf]

2. Materials and methods

2.2 Experimental design

Animal husbandry and experimental procedures were in accordance with the EU Directive 2010/63/EU for animal experiments and approved by the national ethical committee for the care and use of animals (approval ID 3136). Every effort was made to minimize suffering and the number of animals used by respecting the principle of the 3Rs (replacement, reduction, and refinement). Seven-week-old male mice (Balb/c [a highly stress-sensitive strain]; Janvier Labs, Le Genest Saint-Isle, France) were housed under a normal 12-h light/dark cycle (07 h - 19 h) with food and water available *ad libitum* in a controlled environment (22 ± 1°C, 40% humidity). Mice were handled daily for one week before starting the experiment to minimize stress reactions to manipulation. Mice were fed an A04 diet (Safe, Augy, France). They were supplemented by gavage (feeding probe V0105040, ECIMED, Boissy-Saint-Léger, France) with fish hydrolysate (300 mg/kg) or water, for the control group, each morning for seven days. On the last day of supplementation, half of the mice (n = 8/group) of each group (supplemented with fish hydrolysate or control) were subjected to an acute mild stress. The stress protocol consisted of submitting the mice to an open field (OF) test for 10 min immediately followed by an elevated plus maze (EPM) test for 5 min. Both tests induce mild stress in animals by placing them in an anxiogenic condition. Mice were euthanized by decapitation after 5% isoflurane inhalation 90 min following the stress protocol (t90 min). Hippocampi were collected and frozen at -80°C (Figure 1).

2.3 Label-free quantitative proteomics

2.3.2 Nanoliquid chromatography coupled with tandem mass spectrometry (NanoLC-MS/MS)

Approximately 200 ng of the resulting peptide mixtures were separated on a 75 µm × 250 mm IonOpticks Aurora 2 C18 column (Ion Opticks Pty Ltd., Bundoora, Australia). A gradient of reversed-phase solvents (Solvent A: 0.1% formic acid, 98% H_2O MilliQ, 2% acetonitrile; Solvent B: 0.1% formic acid, 100% acetonitrile) was run on a NanoElute HPLC System (Bruker Daltonik GmbH, Bremen, Germany) at a flow rate of 400 nL/min at 50°C. The liquid chromatography (LC) run lasted for 120 min (2% to 15% solvent B over 60 min, up to 25% at 90 min, up to 37% at 100 min, up to 95% at 110 min, and finally 95% for 10 min to wash the column). The column was coupled online to a TIMS TOF Pro (Bruker Daltonik) instrument with a CaptiveSpray ion source (Bruker Daltonik). The temperature of the ion transfer capillary was set to 180°C. Ions were allowed to accumulate for 114 ms and mobility separation was achieved by ramping the entrance potential from −160 V to −20 V within 114 ms. The MS and MS/MS mass spectra were acquired with average resolutions of 60,000 and 50,000 full width at half maximum (mass range 100–1700 m/z), respectively. To enable the PASEF method, precursor m/z and mobility information was first derived from full-scan TIMS-MS experiments (with a mass range of m/z 100–1700). The quadrupole isolation width was set to 2 and 3 Th, and, for fragmentation, the collision energies varied between 31 and 52 eV, depending on the precursor mass and charge. TIMS, MS operation, and PASEF were controlled and synchronized using the control instrument software OtofControl 6.2 (Bruker Daltonik). LC-MS/MS data were acquired using the PASEF method, with a total cycle time of 1.28 s, including 1 TIMS MS scan and 10 PASEF MS/MS scans. Ion mobility-resolved mass spectra and nested ion mobility vs. m/z distributions, as well as the summed fragment ion intensities, were extracted from the raw data file using DataAnalysis 5.3 (Bruker Daltonik).
